# Correction: Nazarloo et al. Oxytocin, Vasopressin and Stress: A Hormetic Perspective. *Curr. Issues Mol. Biol.* 2025, *47*, 632

**DOI:** 10.3390/cimb48030246

**Published:** 2026-02-26

**Authors:** Hans P. Nazarloo, Marcy A. Kingsbury, Hannah Lamont, Caitlin V. Dale, Parmida Nazarloo, John M. Davis, Eric C. Porges, Steven P. Cuffe, C. Sue Carter

**Affiliations:** 1 Department of Psychiatry, College of Medicine-Jacksonville, University of Florida, 580 West 8th St., Tower II, 6th Floor, Jacksonville, FL 32209, USA; steven.cuffe@jax.ufl.edu (S.P.C.); suecarterporges@gmail.com (C.S.C.); 2 Lurie Center for Autism, Mass General Research Institute, Harvard Medical School, Charlestown, MA 02129, USA; makingsbury@mgh.harvard.edu (M.A.K.); hhl23@gsbs.rutgers.edu (H.L.); 3 Dedman College of Humanities and Sciences, Southern Methodist University, Dallas, TX 75205, USA; cate@smu.edu; 4 Traumatic Stress Research Consortium, Kinsey Institute, Indiana University, Bloomington, IN 47405, USA; pnazarl@iu.edu; 5 Department of Psychiatry, University of Illinois at Chicago, Chicago, IL 60607, USA; davisjm@uic.edu; 6 Department of Clinical and Health Psychology, College of Public Health and Health Professions, University of Florida, Gainesville, FL 32611, USA; eporges@ufl.edu; 7 Center for Cognitive Aging and Memory, Evelyn F. and William L. McKnight Brain Institute, University of Florida, Gainesville, FL 32611, USA

The authors would like to make the following correction to their published paper [1]. The change is as follows:

Reference [32,33,56,59,62–65,68,69,71,76,78,84,86,98,102,107,109,113,118,119,123,127,139,140,142,145,147,150,152–154,159,168,169,179,180,182,184–186,193–195] in the original publication is incorrectly cited and needs to be replaced with the following reference:

32. Lee, I.H.; Cao, L.; Mostoslavsky, R.; Lombard, D.B.; Liu, J.; Bruns, N.E.; Tsokos, M.; Alt, F.W.; Finkel, T. A Role for the NAD-Dependent Deacetylase Sirt1 in the Regulation of Autophagy. *Proc. Natl. Acad. Sci. USA* **2008**, *105*, 3374–3379. https://doi.org/10.1073/pnas.0712145105.
33. Hernández-Camacho, J.D.; García-Corzo, L.; Moreno Fernández-Ayala, D.J.; Navas, P.; López-Lluch, G. Coenzyme Q at the Hinge of Health and Metabolic Diseases. *Antioxidants* **2021**, *10*, 1785. https://doi.org/10.3390/antiox10111785.
56. Sukhareva, E.V. The role of the corticotropin-releasing hormone and its receptors in the regulation of stress response. *Vavilovskii Zhurnal Genet. Selektsii* **2021**, *25*, 216–223. https://doi.org/10.18699/VJ21.025.
59. Bagosi, Z.; Megyesi, K.; Ayman, J.; Rudersdorf, H.; Ayaz, M.K.; Csabafi, K. The Role of Corticotropin-Releasing Factor (CRF) and CRF-Related Peptides in the Social Behavior of Rodents. *Biomedicines* **2023**, *11*, 2217. https://doi.org/10.3390/biomedicines11082217.
62. Sleiman, S.F.; Henry, J.; Al-Haddad, R.; El Hayek, L.; Abou Haidar, E.; Stringer, T.; Ulja, D.; Karuppagounder, S.S.; Holson, E.B.; Ratan, R.R.; et al. Exercise Promotes the Expression of Brain-Derived Neurotrophic Factor (BDNF) through the Action of the Ketone Body β-Hydroxybutyrate. *eLife* **2016**, *5*, e15092. https://doi.org/10.7554/eLife.15092.
63. Watts, J.A.; Arroyo, J.P. Rethinking Vasopressin: New Insights into Vasopressin Signaling and Its Implications. *Kidney360* **2023**, *4*, 1174–1180. https://doi.org/10.34067/KID.0000000000000194.
64. Koshimizu, T.-A.; Nakamura, K.; Egashira, N.; Hiroyama, M.; Nonoguchi, H.; Tanoue, A. Vasopressin V1a and V1b Receptors: From Molecules to Physiological Systems. *Physiol. Rev.* **2012**, *92*, 1813–1864. https://doi.org/10.1152/physrev.00035.2011.
65. Uzar, M.; Dmitrzak-Węglarz, M.; Słopień, A. The Role of Oxytocin and Vasopressin in People with Borderline Personality Disorder: A Closer Look at Adolescents. *Int. J. Mol. Sci.* **2024**, *25*, 12046. https://doi.org/10.3390/ijms252212046.
68. Wan, Y.; Liu, J.; Mai, Y.; Hong, Y.; Jia, Z.; Tian, G.; Liu, Y.; Liang, H.; Liu, J. Current Advances and Future Trends of Hormesis in Disease. *NPJ Aging* **2024**, *10*, 26. https://doi.org/10.1038/s41514-024-00155-3.
69. Guan, G.; Chen, Y.; Dong, Y. Unraveling the AMPK–SIRT1–FOXO Pathway: The In-Depth Analysis and Breakthrough Prospects of Oxidative Stress-Induced Diseases. *Antioxidants* **2025**, *14*, 70. https://doi.org/10.3390/antiox14010070.
71. Perrone, P.; D’Angelo, S. Hormesis and Health: Molecular Mechanisms and the Key Role of Polyphenols. *Food Chem. Adv.* **2025**, *7*, 101030. https://doi.org/10.1016/j.focha.2025.101030.
76. Wang, P.; Yang, H.-P.; Tian, S.; Wang, L.; Wang, S.C.; Zhang, F.; Wang, Y.-F. Oxytocin-Secreting System: A Major Part of the Neuroendocrine Center Regulating Immunologic Activity. *J. Neuroimmunol*. **2015**, *289*, 152–161. https://doi.org/10.1016/j.jneuroim.2015.11.001.
78. Ma, Q. Role of Nrf2 in Oxidative Stress and Toxicity. *Annu. Rev. Pharmacol. Toxicol.* **2013**, *53*, 401–426. https://doi.org/10.1146/annurev-pharmtox-011112-140320.
84. Meyer-Lindenberg, A.; Domes, G.; Kirsch, P.; Heinrichs, M. Oxytocin and Vasopressin in the Human Brain: Social Neuropeptides for Translational Medicine. *Nat. Rev. Neurosci.* **2011**, *12*, 524–538. https://doi.org/10.1038/nrn3044.
86. Aguilera, G.; Subburaju, S.; Young, S.; Chen, J. The Parvocellular Vasopressinergic System and Responsiveness of the Hypothalamic–Pituitary–Adrenal Axis during Chronic Stress. *Prog. Brain Res.* **2008**, *170*, 29–39. https://doi.org/10.1016/S0079-6123(08)00403-2.
98. Theodosis, D.T.; Poulain, D.A.; Oliet, S.H.R. Activity-Dependent Structural and Functional Plasticity of Astrocyte–Neuron Interactions. *Physiol. Rev.* **2008**, *88*, 983–1008. https://doi.org/10.1152/physrev.00036.2007.
102. Aguilera, G.; Rabadan-Diehl, C. Vasopressinergic Regulation of the Hypothalamic–Pituitary–Adrenal Axis: Implications for Stress Adaptation. *Regul. Pept.* **2000**, *96*, 23–29. https://doi.org/10.1016/S0167-0115(00)00196-8.
107. Russo, S.J.; Murrough, J.W.; Han, M.-H.; Charney, D.S.; Nestler, E.J. Neurobiology of Resilience. *Nat. Neurosci*. **2012**, *15*, 1475–1484. https://doi.org/10.1038/nn.3234.
109. Balmus, I.M.; Ciobica, A.; Stoica, B.; Lefter, R.; Cojocaru, S.; Reznikov, A.G. Effects of Oxytocin Administration on Oxidative Markers in the Temporal Lobe of Aged Rats. *Neurophysiology* **2019**, *51*, 18–24. https://doi.org/10.1007/s11062-019-09785-w.
113. Griebel, G.; Simiand, J.; Serradeil-Le Gal, C.; Wagnon, J.; Pascal, M.; Scatton, B.; Maffrand, J.-P.; Soubrie, P. Anxiolytic- and Antidepressant-Like Effects of the Non-Peptide Vasopressin V1b Receptor Antagonist, SSR149415, Suggest an Innovative Approach for the Treatment of Stress-Related Disorders. *Proc. Natl. Acad. Sci. USA* **2002**, *99*, 6370–6375. https://doi.org/10.1073/pnas.092012099.
118. Ulrich-Lai, Y.M.; Herman, J.P. Neural Regulation of Endocrine and Autonomic Stress Responses. *Nat. Rev. Neurosci*. **2009**, *10*, 397–409. https://doi.org/10.1038/nrn2647.
119. Mullur, R.; Liu, Y.-Y.; Brent, G.A. Thyroid Hormone Regulation of Metabolism. *Physiol. Rev.* **2014**, *94*, 355–382. https://doi.org/10.1152/physrev.00030.2013.
123. McClelland, S.; Korosi, A.; Cope, J.; Ivy, A.; Baram, T.Z. Emerging Roles of Epigenetic Mechanisms in the Enduring Effects of Early-Life Stress and Experience on Learning and Memory. *Neurobiol. Learn. Mem.* **2011**, *96*, 79–88. https://doi.org/10.1016/j.nlm.2011.02.008.
127. Myers, B.; McKlveen, J.M.; Herman, J.P. Neural Regulation of the Stress Response: The Many Faces of Feedback. *Cell. Mol. Neurobiol*. **2012**, *32*, 683–694. https://doi.org/10.1007/s10571-012-9801-y.
139. Flaherty, S.E.; Bezy, O.; Zheng, W.; Yan, D.; Li, X.; Jagarlapudi, S.; Albuquerque, B.; Esquejo, R.M.; Peloquin, M.; Semache, M.; et al. Chronic UCN2 Treatment Desensitizes CRHR2 and Improves Insulin Sensitivity. *Nat. Commun.* **2023**, *14*, 3953. https://doi.org/10.1038/s41467-023-39597-w.
140. Dumitras, A.G.; Piccoli, G.; Tellkamp, F.; Keufgens, L.; Baraldo, M.; Zorzato, S.; Cussonneau, L.; Nogara, L.; Krüger, M.; Blaauw, B. Neural Stimulation Suppresses mTORC1-Mediated Protein Synthesis in Skeletal Muscle. *Sci. Adv.* **2025**, *11*, eadt4955. https://doi.org/10.1126/sciadv.adt4955.
142. Sipos, E.; Török, B.; Barna, I.; Engelmann, M.; Zelena, D. Vasopressin and Post-Traumatic Stress Disorder. *Stress* **2020**, *23*, 732–745. https://doi.org/10.1080/10253890.2020.1826430.
145. Heinrichs, M.; Baumgartner, T.; Kirschbaum, C.; Ehlert, U. Social Support and Oxytocin Interact to Suppress Cortisol and Subjective Responses to Psychosocial Stress. *Biol. Psychiatry* **2003**, *54*, 1389–1398. https://doi.org/10.1016/S0006-3223(03)00465-7.
147. Stoop, R. Neuromodulation by Oxytocin and Vasopressin. *Neuron* **2012**, *76*, 142–159. https://doi.org/10.1016/j.neuron.2012.09.025.
150. Inoue, T.; Yamakage, H.; Tanaka, M.; Kusakabe, T.; Shimatsu, A.; Satoh-Asahara, N. Oxytocin Suppresses Inflammatory Responses Induced by Lipopolysaccharide through Inhibition of the eIF-2α–ATF4 Pathway in Mouse Microglia. *Cells* **2019**, *8*, 527. https://doi.org/10.3390/cells8060527.
152. Xu, S.; Qin, B.; Shi, A.; Zhao, J.; Guo, X.; Dong, L. Oxytocin Inhibited Stress-Induced Visceral Hypersensitivity, Enteric Glial Cells Activation, and Release of Proinflammatory Cytokines in Maternal Separated Rats. *Eur. J. Pharmacol*. **2018**, *818*, 578–584. https://doi.org/10.1016/j.ejphar.2017.11.018.
153. Sripada, C.S.; Phan, K.L.; Labuschagne, I.; Welsh, R.; Nathan, P.J.; Wood, A.G. Oxytocin Enhances Resting-State Connectivity between Amygdala and Medial Frontal Cortex. *Int. J. Neuropsychopharmacol*. **2013**, *16*, 255–260. https://doi.org/10.1017/S1461145712000533.
154. Janeček, M.; Dabrowska, J. Oxytocin Facilitates Adaptive Fear and Attenuates Anxiety Responses in Animal Models and Human Studies—Potential Interaction with the Corticotropin-Releasing Factor (CRF) System in the Bed Nucleus of the Stria Terminalis (BNST). *Cell Tissue Res.* **2019**, *375*, 143–172. https://doi.org/10.1007/s00441-018-2889-8.
159. Kanes, S.J.; Dennie, L.; Perera, P. Targeting the Arginine Vasopressin V1b Receptor System and Stress Response in Depression and Other Neuropsychiatric Disorders. *Neuropsychiatr. Dis. Treat.* **2023**, *19*, 811–828. https://doi.org/10.2147/NDT.S402831.
168. Aguilera, G. Regulation of Pituitary ACTH Secretion during Chronic Stress. *Front. Neuroendocrinol*. **1994**, *15*, 321–350. https://doi.org/10.1006/frne.1994.1013.
169. Russell, G.M.; Kalafatakis, K.; Lightman, S.L. The Importance of Biological Oscillators for Hypothalamic–Pituitary–Adrenal Activity and Tissue Glucocorticoid Response: Coordinating Stress and Neurobehavioural Adaptation. *J. Neuroendocrinol*. **2015**, *27*, 378–388. https://doi.org/10.1111/jne.12247.
179. Lightman, S.L.; Conway-Campbell, B.L. The Crucial Role of Pulsatile Activity of the HPA Axis for Continuous Dynamic Equilibration. *Nat. Rev. Neurosci*. **2010**, *11*, 710–718. https://doi.org/10.1038/nrn2914.
180. Dagnino-Subiabre, A. Resilience to Stress and Social Touch. *Curr. Opin. Behav. Sci.* **2021**, *43*, 75–79. https://doi.org/10.1016/j.cobeha.2021.08.011.
182. Quintana, D.S.; Kemp, A.H.; Alvares, G.A.; Guastella, A.J. A Role for Autonomic Cardiac Control in the Effects of Oxytocin on Social Behavior and Psychiatric Illness. *Front. Neurosci.* **2013**, *7*, 48. https://doi.org/10.3389/fnins.2013.00048.
184. Holsboer, F.; Ising, M. Precision Psychiatry Approach to Treat Depression and Anxiety Targeting the Stress Hormone System—V1b Antagonists as a Case in Point. *Pharmacopsychiatry* **2024**, *57*, 263–274. https://doi.org/10.1055/a-2372-3549.
185. Hu, H.; Zarate, C.A., Jr.; Verbalis, J. Arginine Vasopressin in Mood Disorders: A Potential Biomarker of Disease Pathology and a Target for Pharmacologic Intervention. *Psychiatry Clin. Neurosci.* **2024**, *78*, 495–506. https://doi.org/10.1111/pcn.13703.
186. Schoormans, D.; Kop, W.J.; Kunst, L.E.; Riem, M.M.E. Oxytocin Effects on Resting-State Heart Rate Variability in Women: The Role of Childhood Rearing Experiences. *Compr. Psychoneuroendocrinol.* **2020**, *3*, 100007. https://doi.org/10.1016/j.cpnec.2020.100007.
193. Forcina, L.; Franceschi, C.; Musarò, A. The Hormetic and Hermetic Role of IL-6. *Ageing Res. Rev.* **2022**, *80*, 101697. https://doi.org/10.1016/j.arr.2022.101697.
194. Johnson, J.D.; Barnard, D.F.; Kulp, A.C.; Mehta, D.M. Neuroendocrine Regulation of Brain Cytokines After Psychological Stress. *J. Endocr. Soc.* **2019**, *3*, 1302–1320. https://doi.org/10.1210/js.2019-00053.
195. Zhang, B.; Chang, J.Y.; Lee, M.H.; Ju, S.-H.; Yi, H.-S.; Shong, M. Mitochondrial Stress and Mitokines: Therapeutic Perspectives for the Treatment of Metabolic Diseases. *Diabetes Metab. J.* **2024**, *48*, 1–18. https://doi.org/10.4093/dmj.2023.0115.

The authors state that the scientific conclusions are unaffected. This correction was approved by the Academic Editor. The original publication has also been updated.
